# A Case Report of Wernicke's Encephalopathy Associated With Schizophrenia

**DOI:** 10.3389/fpsyt.2021.657649

**Published:** 2021-05-05

**Authors:** Jingqi He, Jinguang Li, Zhijun Li, Honghong Ren, Xiaogang Chen, Jinsong Tang

**Affiliations:** ^1^National Clinical Research Center for Mental Disorders, and Department of Psychiatry, The Second Xiangya Hospital of Central South University, Changsha, China; ^2^Affiliated Wuhan Mental Health Center, Tongji Medical College of Huazhong University of Science and Technology, Wuhan, China; ^3^Department of Psychiatry, Sir Run-Run Shaw Hospital, School of Medicine, Zhejiang University, Hangzhou, China; ^4^Zigong Mental Health Center, Zigong, China

**Keywords:** Wernicke's encephalopathy, schizophrenia, thiamine, non-alcoholic, classic triad

## Abstract

**Introduction:** Wernicke's encephalopathy (WE) is a severe neurological syndrome often associated with alcoholism. Clinicians tend to ignore WE in other non-alcoholic clinical settings related to malnutrition and thiamine deficiency, resulting in delayed diagnosis. The diagnosis becomes more difficult when WE is secondary to psychiatric illnesses as symptoms can be masked by the primary disease.

**Case Presentation:** We present a case of a 56-year-old female patient with schizophrenia who was admitted to the hospital for mental and behavioral disorder, without history of alcohol. She presented symptoms of ophthalmoplegia and high muscular tension, and the brain MRI showed symmetric lesions in the bilateral basal ganglia and third ventricle. She responded well to thiamine and was discharged on hospital day 22.

**Conclusion:** The psychiatrists should be on the alert for starvation-induced WE, especially for patients suffering from malnutrition. WE is a preventable and treatable disease, so once suspected of WE, patients ought to take adequate supplements of thiamine immediately.

## Introduction

Wernicke's encephalopathy (WE) is a medical emergency caused by thiamine deficiency, which is greatly underdiagnosed before death. Clinically, alcoholism is the most common cause of WE. The typical symptoms of WE include mental status change, ocular abnormalities, and motor problems, such as gait incoordination and ataxia. WE can be fatal if it is not managed timely, so early diagnosis and treatment are crucial.

Due to the close link to alcoholism, the diagnosis of non-alcoholic WE is often missed. When it occurs in psychiatric illnesses, the situation will get worse. Since there have been only a few reports about WE-associated schizophrenia (1–3), we report a case of non-alcoholic WE in a patient with chronic schizophrenia whose negative symptoms were prominent.

## Case Presentation

A 56-year-old female patient with a pertinent history of schizophrenia, primary angle-closure glaucoma with the left eye, chronic bronchitis, coronary heart disease, and renal calculi was brought to the emergency department with altered mental status and mild respiratory disorder. Three months ago, after drug withdrawal without the guidance of doctors, she began to have near-total absence of motion and speech and refused to take antipsychotics and food. Even though she had been hospitalized in another hospital a month earlier and given antipsychotics and nutritional support, her condition had not improved. A day earlier, the patient had been noted to open her mouth to breath, followed by the whirring sound. According to her husband, she had no history of alcohol, tobacco, or substance abuse. However, her compliance with antipsychotics was so poor that the control of psychosis was relatively ineffective, and her ability to contact and communicate had been impaired in recent years. Neither she nor her family could provide further details.

Her physical examination revealed that she had a weight of 45 kg, a temperature of 36.4?C, a blood pressure of 129/84 mmHg, and a pulse of 105/min. She was bed-bound, except for relieving herself, clear in consciousness with a blank and blunted facial expression, and refused to answer questions. She was unable to cooperate with most of the physical examinations because she refused to follow instructions. Her bilateral pupils were equal and round. The left pupil was fixed and had no light reflex and the right pupil was reactive to light. Of note is that her left horizontal gaze palsy was obvious, but her eyes were closed tightly most of the time. She presented with increased muscular tension and tendon hyperreflexia in extremity. Other than that, the remainder of the neurologic examination was unremarkable or uncompleted.

Her initial laboratory workup was as follows: white blood cell count, 13.03 × 10^3^/μL with 88.80% neutrophils; C-reactive protein, 21.2 mg/L; procalcitonin, 0.31 ng/L; myoglobin, 81 μg/L; and ischemia-modified albumin, 57.6 μ/ml; electrolytes, the level of thyroid hormones, and liver and renal function tests were otherwise unremarkable. Her plasma lactate level was 2.24 mmol/L, which increased very slightly. Chest CT showed diffuse infiltration in her right middle lobe and inferior lobes of bilateral lungs. Her electrocardiogram and brain CT were normal. The respiratory consultant diagnosed her with aspiration pneumonia caused by prolonged bed rest and improper breathing pattern.

She was admitted to the psychiatry department at first, with nasogastric feeding. No antipsychotics were applied because of the diagnosis of malignant syndrome that needs to be ruled out. After being given oral and parenteral nutrition for 3 days, she seemed to get better. She removed the nasogastric tube by herself for feeling uncomfortable and ate a little porridge. She could complete some simple instructions like “drink water” and the whirring sound associated with breath became less. While on hospital day 5, the patient suddenly had difficulty in breathing and became confused. Arterial blood gas demonstrated type I respiratory failure with a partial pressure of carbon dioxide of 41.60 mmHg and a partial pressure of oxygen of 53.60 mmHg. Saturation of blood oxygen fluctuated between 63 and 83%, increasing immediately after airway suction. The respiratory consultant explained that the phenomenon was a result of a massive airway secretion produced through mouth breathing.

**Figure 1:**
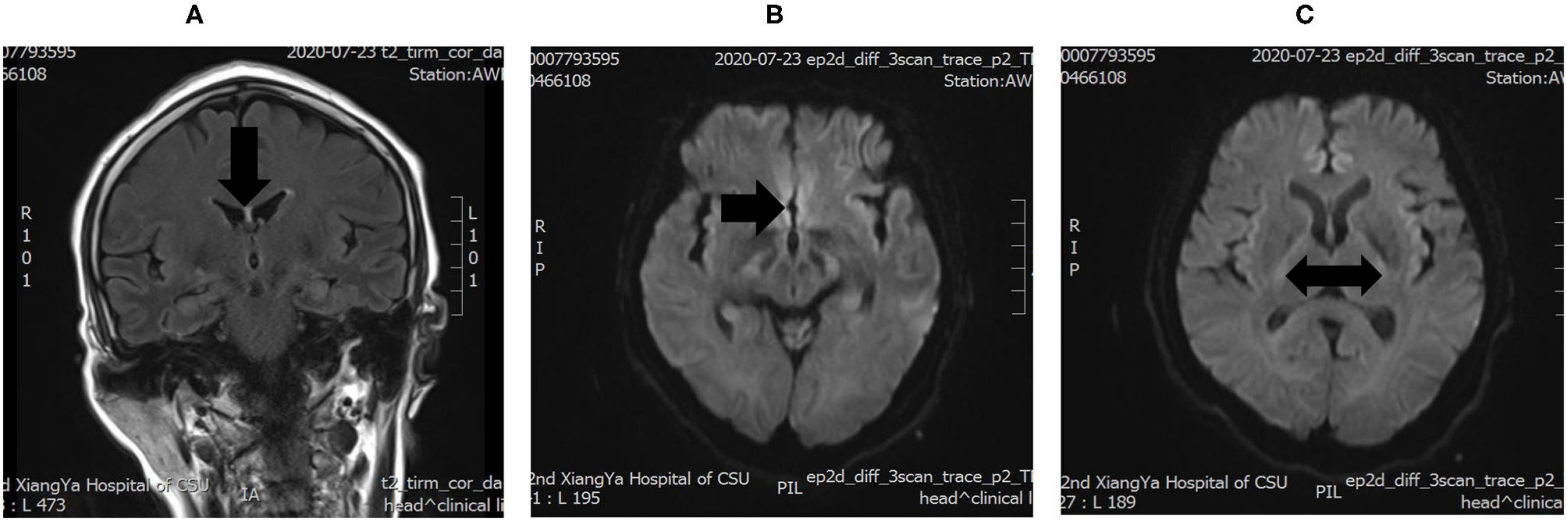
**(A)** increased T2 signals in the periventricular region around the third ventricle. **(B,C)** Diffusion-weighted imaging (DWI) showing Wernicke's encephalopathy (WE) changes in the bilateral basal ganglia.

On hospital day 6, the patient was eventually transferred to the neurology intensive care unit and a lumbar puncture and a brain MRI were performed. Cerebrospinal fluid (CSF) biomarkers were normal, except for a slightly elevated glucose (5.51 mmol/L). The brain MRI showed symmetrical increased T2 signal and restricted diffusion in the bilateral basal ganglia and around the third ventricle (Figure 1), which confirmed the diagnosis of WE.

The patient was treated for WE with 100 mg of thiamine injected intramuscularly, three times a day and 100 mg/day by nasal feeding once the diagnosis was clear. She was also given 13.5 g/day piperacillin–tazobactam intravenously for anti-infective therapy and sulpiride for her schizophrenia. On hospital day 18, she regained consciousness and was able to answer some simple questions and recall the daily meals. On hospital day 27, she improved and was discharged without obvious sequelae. Six months after discharge, we followed up with her by telephone visit and were told that she was able to do some simple housework with no significant memory disorders; unfortunately, she responded poorly to antipsychotics because the choice of antipsychotics was limited by her glaucoma.

## Discussion

The fact that our patient had multiple underlying diseases and accompanying symptoms and the lack of evidence of alcoholism made the diagnosis of WE difficult. Her altered mental status and increased muscular tension tended to be explained by primary mental disease or side effects caused by antipsychotics rather than WE at first. Although her physical examination showed horizontal gaze, which was one of the common signs of WE, it was overlooked due to severe respiratory disorder and significant psychiatric symptoms. Considering her history of food refusal and unremarkable laboratory studies, the diagnosis of WE was suggested. Finally, the effective response to thiamine and MRI scan confirmed the primary diagnosis of WE.

WE is a serious neurological disorder resulting from thiamine deficiency with a high mortality of 17% (4). The disease was first described by Carl Wernicke in 1881, characterized by the classic triad of altered mentation, gait ataxia, and ocular signs (5). Alcoholism is the most common clinical setting related to WE; other clinical settings like malignant disease, gastrointestinal disease and surgery, and vomiting due to hyperemesis gravidarum have been recognized in recent years (6). According to previous autopsy studies, up to 94% of non-alcoholic patients were undiagnosed during life due to a close association with alcoholism, much higher than in alcoholic patients (68%) (6).

Thiamine plays a crucial role in maintaining the proper function of the nervous, cardiovascular, and locomotive systems (7). Thiamine pyrophosphate (TPP), the biologically active form of thiamine, is an essential coenzyme of many key enzymes in glucose metabolism, including the tricarboxylic acid (TCA) cycle and the pentose phosphate pathway. Thiamine deficiency can lead to lactic acid accumulation through anaerobic respiration, then brain cytotoxic edema and vasogenic edema develop in vulnerable and sensitive regions (8–10). As a water-soluble vitamin, thiamine is not synthesized by the body and must be obtained from the diet. Normally, the body's stores of thiamine are only sufficient for up to 18 days (11). Thus, dietary thiamine supplements are necessary in daily intake, and the daily requirement is between 1 and 2 mg, related to the carbohydrate intake (12). On the one hand, our patient lived on polished white rice, a staple in the diet of rural Chinese containing less thiamine, which indicates a possibly inadequate thiamine intake in her diet even at the remission phase of schizophrenia. On the other hand, her history of food refusal exposed her to the risk of thiamine deficiency, and thiamine supplement was overlooked before carbohydrate supplement. In addition, previous studies show that there is a peculiar, acquired dysfunction of carbohydrate metabolism in psychiatric patients, often associated with the gut microbiome and the side effects of antipsychotics, which may have made the condition of our patient worse. Casanova reported a schizophrenic patient who developed WE confirmed by autopsy, although she was taken good care of by her family, without a history of malabsorption or alcoholism (13).

WE is commonly elicited by severe, short-term thiamine deficiency (14). The early symptoms of thiamine deficiency are non-specific and varied, such as frequent headaches, fatigue, irritability, abdominal discomfort, and a decline in the growth rate of children, which are similar to the negative symptoms of schizophrenia (lack of interest, apathy, and tiredness) (14–16). WE associated with schizophrenia often happens after rapid loss of weight (16). However, although WE is a clinical diagnosis, the full triad only appears in 16% of patients, and the occurrence seems to be higher in alcoholics (6). Other uncommon signs include stupor, hypotension and tachycardia, hypothermia, bilateral visual disturbances and papilloedema, epileptic seizures, hearing loss and hallucinations, and behavioral disturbances (14). The non-specificity and diversity of the clinical presentations make the diagnosis of WE a great challenge. Furthermore, just as in our patient, patients are unable to cooperate with the neurologic examination, in which case some important signs will not be noticed, which makes an already difficult diagnosis even worse. Guidelines from the European Federation of Neurological Societies recommend that the clinical diagnosis of WE in both alcoholics and non-alcoholics requires two of the following four signs; (i) dietary deficiencies, (ii) eye signs, (iii) cerebellar dysfunction, and (iv) either an altered mental state or mild memory impairment (6).

As a clinical diagnosis, physical examination is important in the early diagnosis of WE. Looking back on our case, though our patient was incapable of cooperating with most of the physical examination, her left gaze palsy was obvious, which was regretfully overlooked at first. As a matter of fact, ocular motor signs like nystagmus, gaze palsy, sixth nerve palsy, etc., are “the earliest signs,” and horizontal gaze-evoked nystagmus is the most common neurological manifestation in patients with thiamine deficiency (4). According to Oudman's relevant systematic review, 80% of patients developing WE associated with schizophrenia showed ocular abnormalities (16). In addition, abnormal vestibulo-ocular reflex can be presented in the early pre-encephalopathy stage of thiamine deficiency (17, 18). Kattah's study showed that the above symptoms could be improved quickly through thiamine supplementation (17, 18). Hence, in the early stage of thiamine deficiency before the development of WE, a careful ocular motor and vestibular examination can be a useful and low-cost tool for rapid diagnosis, and clinicians should be more sensitive to the ocular motor signs during the process of diagnosis (17–19).

Generally, except that protein in CSF can increase in the late stages, laboratory tests have no useful result. Some methods for determining thiamine levels in serum, plasma, or whole blood, such as high-performance liquid chromatography, can be used in the early diagnosis, but remain to be improved and promoted (10, 14, 20). On account of the limited technology, there are some difficulties in popularizing blood thiamine level tests, especially in developing countries. Compared with a laboratory test, imaging studies reveal the advantage. MRI is currently considered the most valuable auxiliary examination for WE. Typical MRI findings include symmetrical alterations in the thalami, mammillary bodies, tectal plate, and periaqueductal area, while the atypical findings include symmetric alterations of the cerebellum, vermis of the cerebellum, cranial nerve nuclei, red nuclei, dentate nuclei, caudate nuclei, splenium, and the cerebral cortex, which are more frequently present in non-alcoholics (21). Diffusion-weighted imaging (DWI), MR spectroscopy, and other applications of MRI may provide meaningful information for the early diagnosis of WE, but this is still unclear at present (21, 22).

Wernicke's encephalopathy is a medical emergency with substantial morbidity and mortality, but it is also easily reversible, so every suspected patient should receive treatment of thiamine supplementation immediately (8, 23). The optimal dose, route, and treatment time have not been determined yet, but it is generally acknowledged that high- and multiple-dose administration is recommended because of the short half-life of thiamine (96 min or less) (23). For example, the European Federation of Neurological Societies guidelines recommend that thiamine should be given intravenously at 200 mg three times daily (6). The Royal College of Physicians suggests that an intramuscular dose of >500 mg of thiamine is required for the first 3 days (24, 25). Non-alcoholics may need a lower dose than alcoholics (10, 14). It is worth mentioning that thiamine should be initiated before carbohydrate supplement; otherwise, glucose will aggravate WE (14). It is clear that inadequate doses of thiamine administration will lead to Korsakoff's syndrome or even death (25). In this case, we treated the patient with a lower dose (400 mg/day), considering her low body weight. In fact, we found an interesting phenomenon when consulting and summarizing related case reports on non-alcoholic WE occurring in China: that the dose of intramuscular thiamine ranges from 100 to 300 mg/day, which was relatively lower than the recommended dosage in Western countries (26–30). We deduce that the reasons for the difference may be racial differences; further evidence-based medical research is needed.

## Conclusion

Food refusal is a common symptom of psychiatric diseases, which can appear in anorexia nervosa, major depression, schizophrenia, etc. In these situations, patients are at high risk of thiamine deficiency and may go on to develop WE. In individuals with schizophrenia, rapid loss of weight can be a signal of thiamine deficiency before the development of WE (16). However, there are a limited numbers of reports about WE in psychiatric patients so far (16). As psychiatrists, we probably will not miss the diagnosis of WE and timely thiamine supplement when we treat alcoholics, but we are likely to overlook non-alcoholic WE, especially when some important mental symptoms and neurological signs of WE can be masked by the patient's primary mental illnesses. Hence, it is important for us to raise awareness of non-alcoholic WE in order to take measures without delay. To reduce mortality and improve prognosis, a detailed neurological examination, early brain MRI, and adequate thiamine supplementation are essential.

## Data Availability Statement

The original contributions presented in the study are included in the article/supplementary material, further inquiries can be directed to the corresponding author/s.

## Ethics Statement

Written informed consent was obtained from the individual(s) for the publication of any potentially identifiable images or data included in this article.

## Author Contributions

JH collected the history and wrote the manuscript with ZL. JL and HR performed manuscript revision. JT and XC reviewed the diagnostic results and contributed to manuscript preparation. All authors contributed to the article and approved the submitted version.

## Conflict of Interest

The authors declare that the research was conducted in the absence of any commercial or financial relationships that could be construed as a potential conflict of interest.
